# p38 phosphorylation in medullary microglia mediates ectopic orofacial inflammatory pain in rats

**DOI:** 10.1186/s12990-015-0053-y

**Published:** 2015-08-12

**Authors:** Masaaki Kiyomoto, Masamichi Shinoda, Kuniya Honda, Yuka Nakaya, Ko Dezawa, Ayano Katagiri, Satoshi Kamakura, Tomio Inoue, Koichi Iwata

**Affiliations:** Department of Oral Physiology, Showa University School of Dentistry, 1-5-8 Hatanodai, Shinagawa-ku, Tokyo, 142-8555 Japan; Department of Physiology, Nihon University School of Dentistry, 1-8-13 Kandasurugadai, Chiyoda-ku, Tokyo, 101-8310 Japan; Department of Oral Diagnostic Sciences, Nihon University School of Dentistry, 1-8-13 Kandasurugadai, Chiyoda-ku, Tokyo, 101-8310 Japan

**Keywords:** Microglia, Ectopic pain, Trigeminal spinal nucleus, Interleukin-1β, Trapezius muscle inflammation

## Abstract

**Background:**

Orofacial inflammatory pain is likely to accompany referred pain in uninflamed orofacial structures. The ectopic pain precludes precise diagnosis and makes treatment problematic, because the underlying mechanism is not well understood. Using the established ectopic orofacial pain model induced by complete Freund’s adjuvant (CFA) injection into trapezius muscle, we analyzed the possible role of p38 phosphorylation in activated microglia in ectopic orofacial pain.

**Results:**

Mechanical allodynia in the lateral facial skin was induced following trapezius muscle inflammation, which accompanied microglial activation with p38 phosphorylation and hyperexcitability of wide dynamic range (WDR) neurons in the trigeminal spinal subnucleus caudalis (Vc). Intra-cisterna successive administration of a p38 mitogen-activated protein kinase selective inhibitor, SB203580, suppressed microglial activation and its phosphorylation of p38. Moreover, SB203580 administration completely suppressed mechanical allodynia in the lateral facial skin and enhanced WDR neuronal excitability in Vc. Microglial interleukin-1β over-expression in Vc was induced by trapezius muscle inflammation, which was significantly suppressed by SB203580 administration.

**Conclusions:**

These findings indicate that microglia, activated via p38 phosphorylation, play a pivotal role in WDR neuronal hyperexcitability, which accounts for the mechanical hypersensitivity in the lateral facial skin associated with trapezius muscle inflammation.

## Background

Referred pain originating from the trapezius muscle frequently produces orofacial pathological pain such as tension-type headaches or temporomandibular disorder (TMD) [1, 2]. Clinically, orofacial pain is likely to occur in areas far away from the trapezius muscle, which causes misdiagnosis and/or inappropriate treatment [3]. In animal experiments, it has been shown that masseter muscle injection of exogenous substances such as glutamate or nerve growth factor produces nociceptive disturbances in discrete areas similar to those reported in TMD patients [4–6]. Nevertheless, the mechanisms underlying nociceptive disturbances in discrete areas associated with such muscle inflammation are still poorly understood.

The mitogen-activated protein kinases (MAPKs), which belong to a highly conserved family of serine/threonine protein kinases, are involved in various cell signaling and gene expression in central nervous system (CNS) [7]. A variety of extracellular stimuli activate intracellular MAPKs by phosphorylation, which modulates intracellular responses driving different downstream signaling [8]. p38, which is a member of a MAPK family, is present constitutively in non-neuronal glial cells in the spinal cord, and is phosphorylated via proinflammatory cytokines released in the spinal cord associated with peripheral inflammation, and is thought to play an essential role in inflammatory pain [9, 10]. Among non-neuronal glial cells in the CNS, microglia, which operates as resident macrophages, are capable of activation following peripheral inflammation. Activated microglia exhibit a morphological change from a ramified shape to an amoeboid shape and an increase in proliferation, which can be defined immunohistochemically using ionized calcium-binding adaptor molecule-1 (Iba1) antibody [11, 12]. By extension, it has been reported that such morphological changes in microglia is caused via the p38 signaling cascades [13, 14]. After microglial activation (phosphorylation), p38 phosphorylation is likely to promote the synthesis of several downstream molecules such as cyclooxygenase-2 or interleukin (IL)-1β in activated microglia via transcriptional regulation [15, 16]. Accordingly, the microglial activation is presumably involved in the generation of central sensitization via production of proinflammatory cytokines, thus contributing to pathological pain.

In this study, we demonstrated the possible role of p38 phosphorylation in activated microglia in ectopic orofacial pain associated with trapezius muscle inflammation using the ectopic orofacial pain model established by complete Freund’s adjuvant (CFA) injection into trapezius muscle [17]. We examined changes in the Iba1 immunoreactivity in trigeminal spinal subnucleus caudalis (Vc). We also considered whether p38 in activated microglia was phosphorylated and whether the inhibition of p38 phosphorylation in microglia depressed mechanical hypersensitivity in facial skin. Moreover, we examined the changes in wide dynamic range (WDR) neuronal excitability due to the inhibition of p38 phosphorylation in microglia following trapezius muscle inflammation.

## Results

### Changes in mechanical sensitivity following CFA injection

**Fig. 1:**
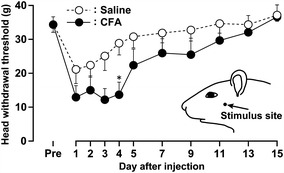
Changes in mechanical sensitivity following CFA injection into trapezius muscle. HWT measured in facial skin for 15 days after CFA or saline injection into the ipsilateral trapezius muscle. The *inset* indicates the stimulus site. *p < 0.05 compared with saline-injected rats. Data represent mean ± SEM. (n = 10 in each group; two-way ANOVA with repeated measures, followed by Bonferroni’s multiple-comparison tests).

HWT to mechanical stimulation of the lateral facial skin significantly decreased on day 4 after CFA injection into the trapezius muscle (13.7 ± 3.7 g) compared with saline-injected rats (28.9 ± 3.5 g) (*p* < 0.05) (Fig. 1). No significant changes in HWT to mechanical stimulation of the contralateral lateral facial skin in the CFA-injected rats were observed compared with naive rats (data not shown). Body weight over the course of the experiment was not significantly different between saline-injected and CFA-injected rats.

### Microglial activation in Vc

**Fig. 2:**
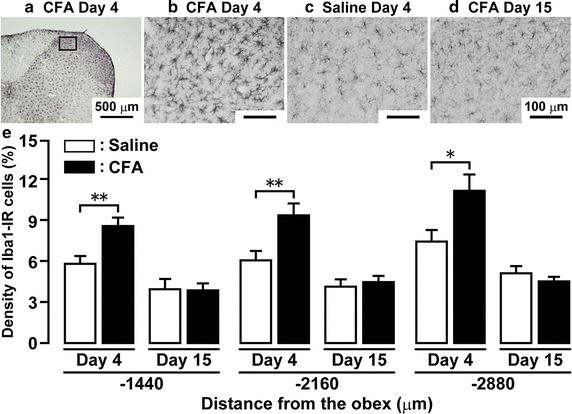
Microglial activation in Vc following CFA injection into trapezius muscle. **a** Photomicrograph of Iba1-IR cells in Vc on day 4 after the CFA injection. High-magnificated photomicrograph of Iba1-IR cells in Vc on day 4 after CFA (**b**) or saline (**c**) injection. **d** High-magnificated photogmicrograph of Iba1-IR cells in Vc on day 15 after CFA injection. *Scale bar* 500 μm (**a**); 100 μm (**b–d**). **e** Density of the Iba1 immuno-products in Vc (1,440, 2,160 and 2,800 μm caudal to the obex) on day 4 and 15 after saline or CFA injection. Data represent mean ± SEM; n = 13–14 in each; *p < 0.05, **p < 0.01; compared to saline-injected rats by Student’s t tests.

Microglia in Vc showed Iba1 immunoreactivity which had a large soma with thick processes on day 4 after CFA injection into the trapezius muscle compared with that of the saline group (Fig. 2a–c). On day 15 after CFA injection, microglia in Vc had no large soma with thick processes (Fig. 2d). The density of Iba1-IR cells on day 4 after CFA injection was much larger than that of saline in Vc at 1,440, 2,160 or 2,880 μm caudal to the obex (−1,440 μm, CFA: 8.7 ± 0.6 %, saline: 5.9 ± 0.5 %; −2,160 μm, CFA: 9.5 ± 0.8 %, saline; 6.2 ± 0.7 %, −2,880 μm, CFA; 11.4 ± 1.2 %, saline: 7.6 ± 0.8 %) (Fig. 2e). On day 15, there were no significant differences in the density of Iba1-IR cells at 1,440, 2,160 or 2,880 μm caudal to the obex between CFA-injected and saline-injected rats.

### p38 phosphorylation in activated microglia in Vc

**Fig. 3:**
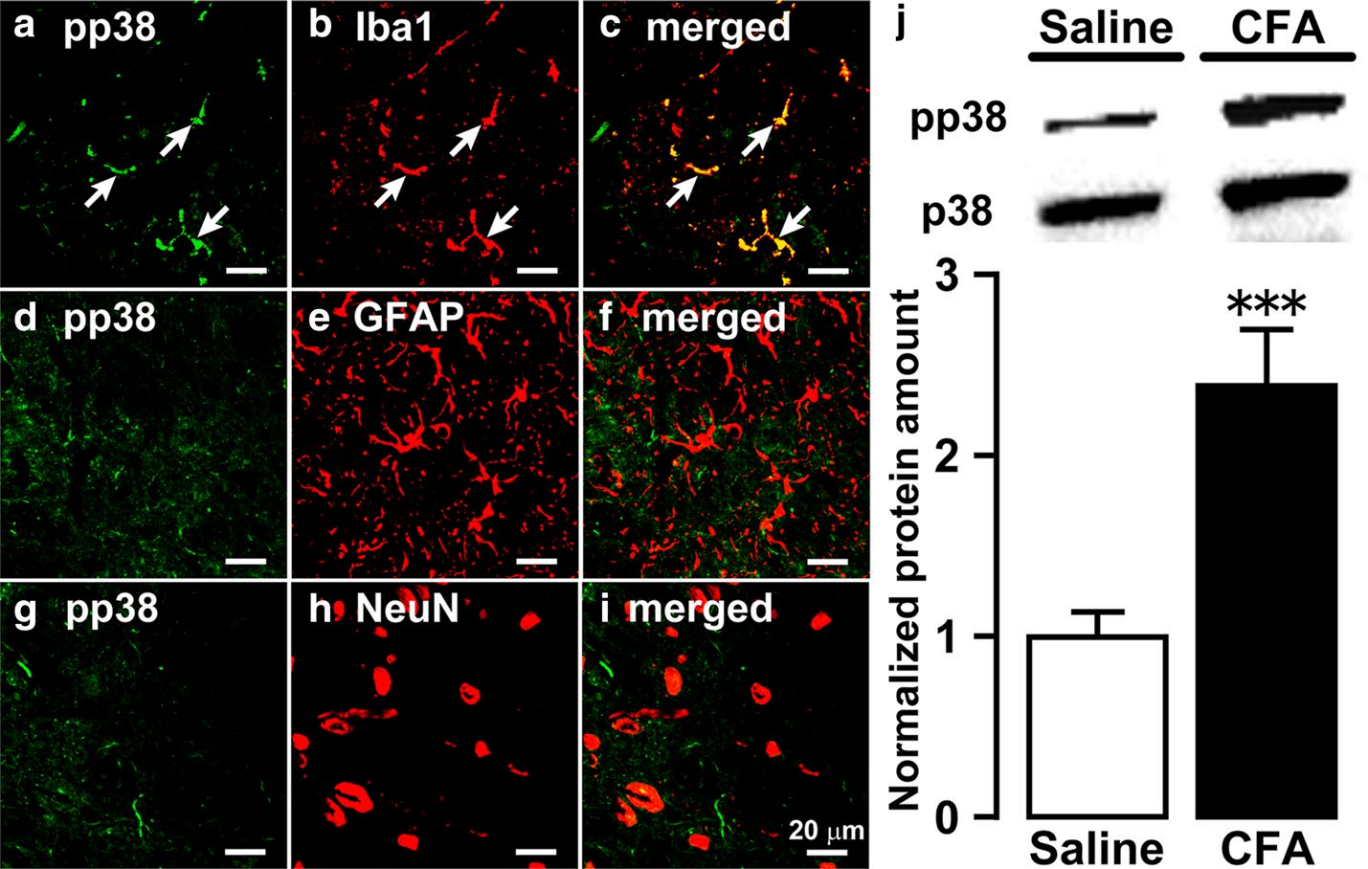
p38 phosphorylation in microglia in Vc. Photomicrographs of pp38-IR cells (**a**, **d**, **g**), Iba1-IR cells (**b**), pp38-IR and Iba1-IR cells (**c**), GFAP-IR cells (**e**), pp38-IR and GFAP-IR cells (**f**), NeuN-IR cells (**h**), and pp38-IR and NeuN-IR cells (**i**) in Vc on day 4 after CFA injection. The* arrow* denotes double-IR cells. **j** Relative amount of pp38 protein in Vc on day 4 after CFA or saline injection. p38 protein was used as a loading control. Data represent mean ± SEM; n = 12 in each; ***p < 0.001; compared to saline-injected rats by Student’s t tests.

The p38 phosphorylation in Vc was examined on day 4 after CFA or saline injection into the trapezius muscle. Iba1-IR cells showed pp38 immunoreactivity in Vc but not in GFAP-IR or NeuN-IR cells (Fig. 3a–i). Moreover, changes in pp38 protein expression in Vc on day 4 after CFA or saline injection were also examined. The normalized protein amount of pp38 in Vc ipsilateral to the CFA injection (2.4 ± 0.3) was significantly greater than that of saline injection (1.0 ± 0.1) (Fig. 3j).

### Effect of i.c.m. SB203580 on mechanical sensitivity and microglial activation

**Fig. 4:**
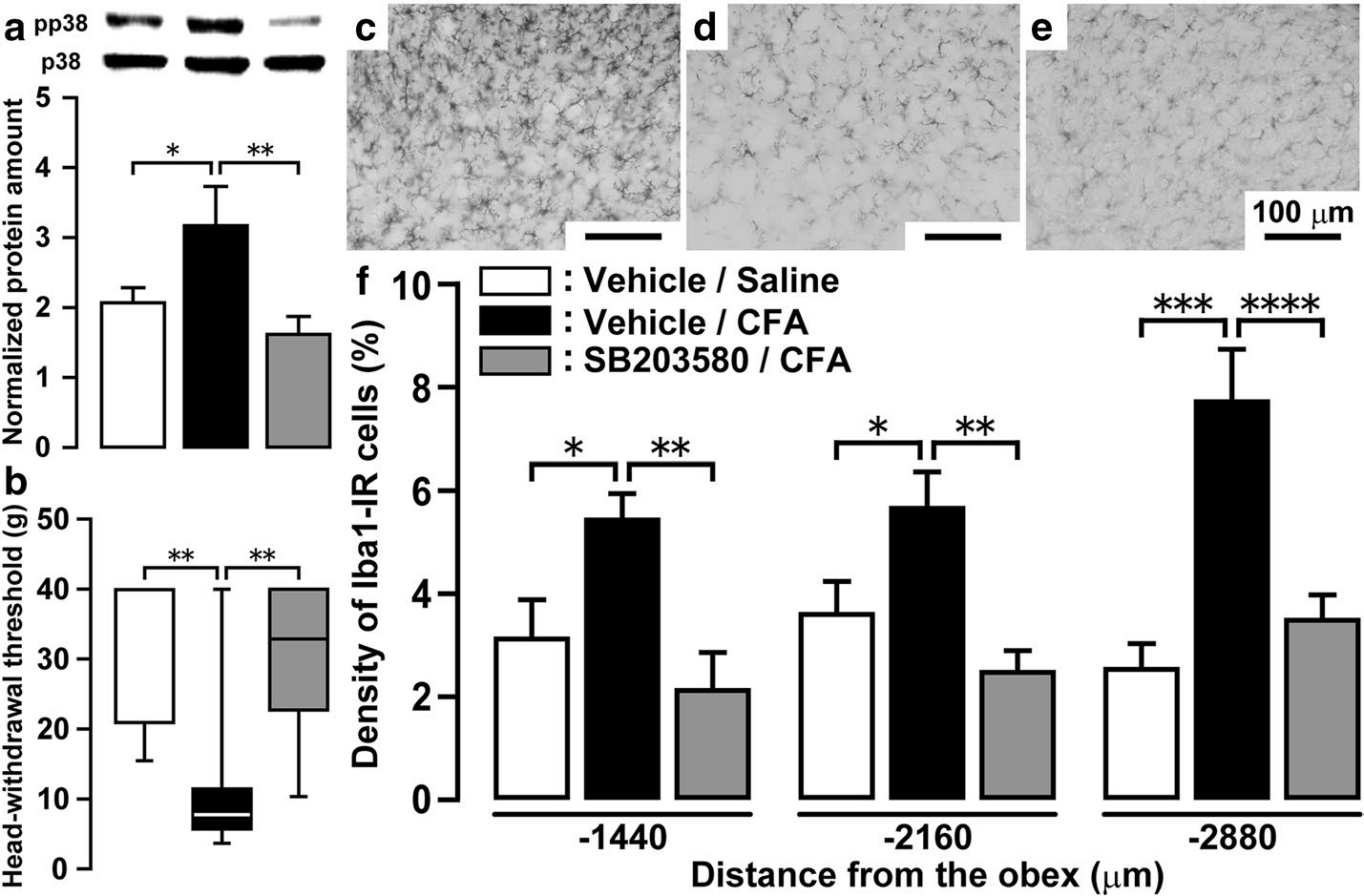
Mechanical sensitivity in facial skin and microglial activation in CFA-injected rats with i.c.m. SB203580 administration. **a** Relative amount of pp38 protein in Vc on day 4 in saline or CFA-injected rats with i.c.m. vehicle or SB203580 administration. p38 protein was used as loading control. Data represent mean ± SEM. n = 16–17 in each. *p < 0.05, **p < 0.01; by one-way ANOVA followed by Tukey’s multiple-comparison tests. **b** HWT measured in facial skin on day 4 after CFA or saline injection with i.c.m. vehicle or SB203580 administration. n = 9–10 in each. **p < 0.01; by one-way ANOVA followed by a Kruskal–Wallis tests. Photomicrograph of Iba1-IR cells on day 4 after CFA injection into the trapezius muscle with i.c.m. vehicle (**c**) or SB203580 (**d**) administration. **e** Photomicrograph of Iba1-IR cells on day 4 after saline injection into the trapezius muscle with i.c.m. vehicle administration. *Scale bar* 100 μm. **f** Density of Iba1 immuno-products in Vc on day 4 in CFA- or saline-injected rats with i.c.m. vehicle or SB203580 administration. Data represent mean ± SEM. n = 9–10 in each. *p < 0.05, **p < 0.01, ***p < 0.001, ****p < 0.0001; by one-way ANOVA followed by Tukey’s multiple-comparison tests.

The normalized protein amount of pp38 in Vc was significantly increased on day 4 following CFA injection with i.c.m. vehicle administration compared with saline-injected rats with i.c.m. vehicle administration, and this increase was completely abolished by successive i.c.m. SB203580 administration (day 0 through day 4) (Fig. 4a). The decrease of HWT on day 4 after CFA injection was also completely abolished by continuous i.c.m. SB203580 administration from day 0 through day 4 (Saline with i.c.m. vehicle, 31.3 ± 3.7 g; CFA with i.c.m. vehicle, 11.3 ± 3.3 g; CFA with i.c.m. SB203580, 30.3 ± 3.6 g) (Fig. 4b). Body weight over the course of the experiment was not significantly different between saline-injected and CFA-injected rats.

On day 4, though Iba1-IR cells in Vc showed large soma with thick processes following CFA injection with i.c.m. vehicle administration, Iba1-IR cells showed no histological changes following CFA injection with i.c.m. SB203580 administration or saline injection with i.c.m. vehicle administration (Fig. 4c–e). Moreover, the increase in the density of Iba1-IR cells in Vc was significantly suppressed by i.c.m. SB203850 administration in CFA-injected rats at 1,440, 2,160 and 2,880 μm caudal to the obex (Fig. 4f).

### Effect of i.c.m. SB203580 on IL-1β release from microglia in Vc

**Fig. 5:**
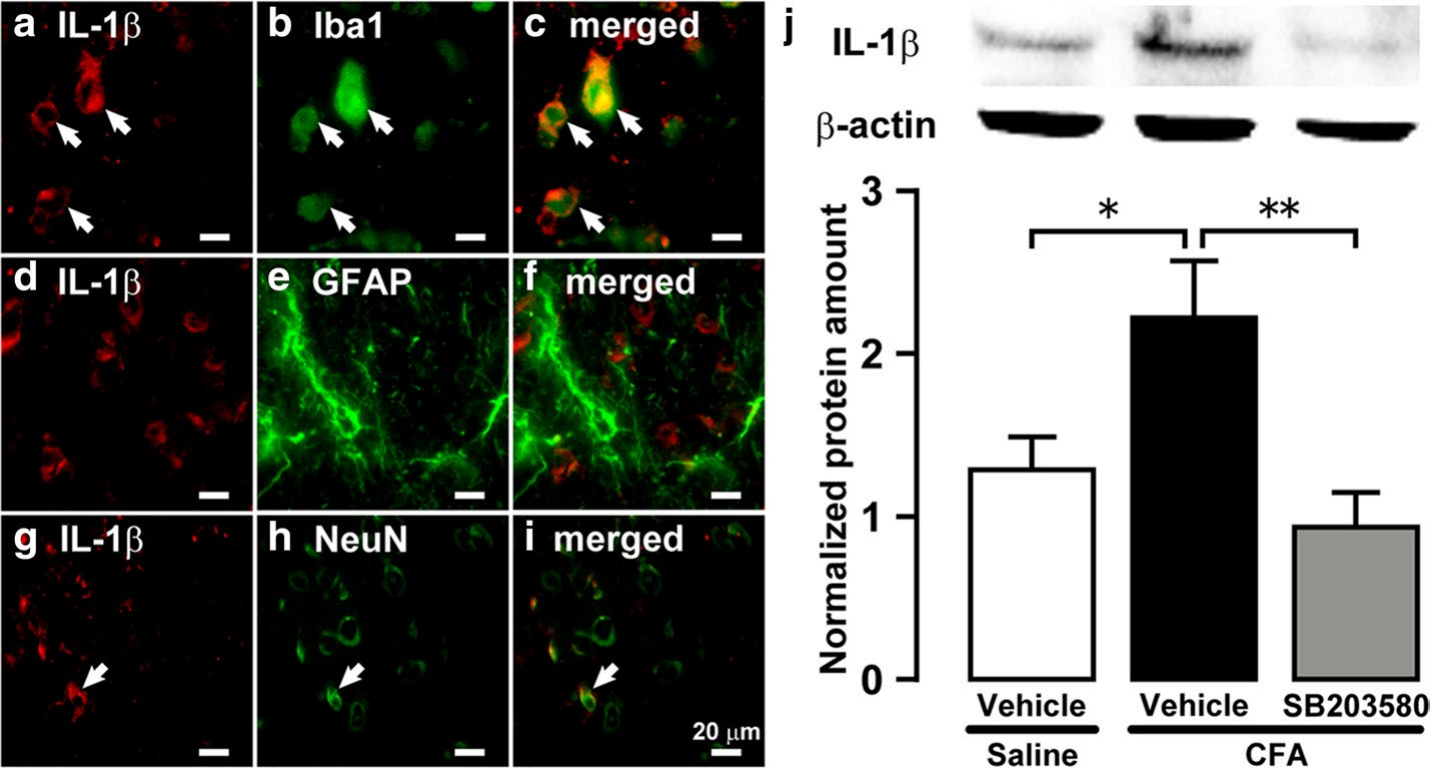
IL-1β protein in saline- or CFA-injected rats with i.c.m. vehicle or SB203580 administration. Photomicrographs of IL-1β-IR cells (**a**, **d**, **g**), Iba1-IR cells (**b**), IL-1β-IR and Iba1-IR cells (**c**), GFAP-IR cells (**e**), IL-1β-IR and GFAP-IR cells (**f**), NeuN-IR cells (**h**), and IL-1β-IR and NeuN-IR cells (**i**) in Vc on day 4 after CFA injection. The *arrow* indicates double-IR cells. **j** Relative amount of IL-1β protein in Vc on day 4 after CFA or saline injection with i.c.m. vehicle or SB203580 administration. β-Actin protein was used as loading control. Data represent mean ± SEM. n = 13 in each. *p < 0.05, **p < 0.01; by one-way ANOVA followed by Tukey’s multiple-comparison tests.

On day 4 after CFA injection into the trapezius muscle, Iba1-IR cells showed IL-1β immunoreactivity in Vc, and several NeuN-IR cells also showed IL-1β immunoreactivity (Fig. 5a–i). The protein amount of IL-1β in Vc ipsilateral to CFA injection was significantly greater than that of saline-injected rats, and the increase of IL-1β protein amount in Vc was completely abolished following i.c.m. SB203580 administration (Fig. 5j).

### Effect of SB203580 on Vc WDR neuronal activity

**Fig. 6:**
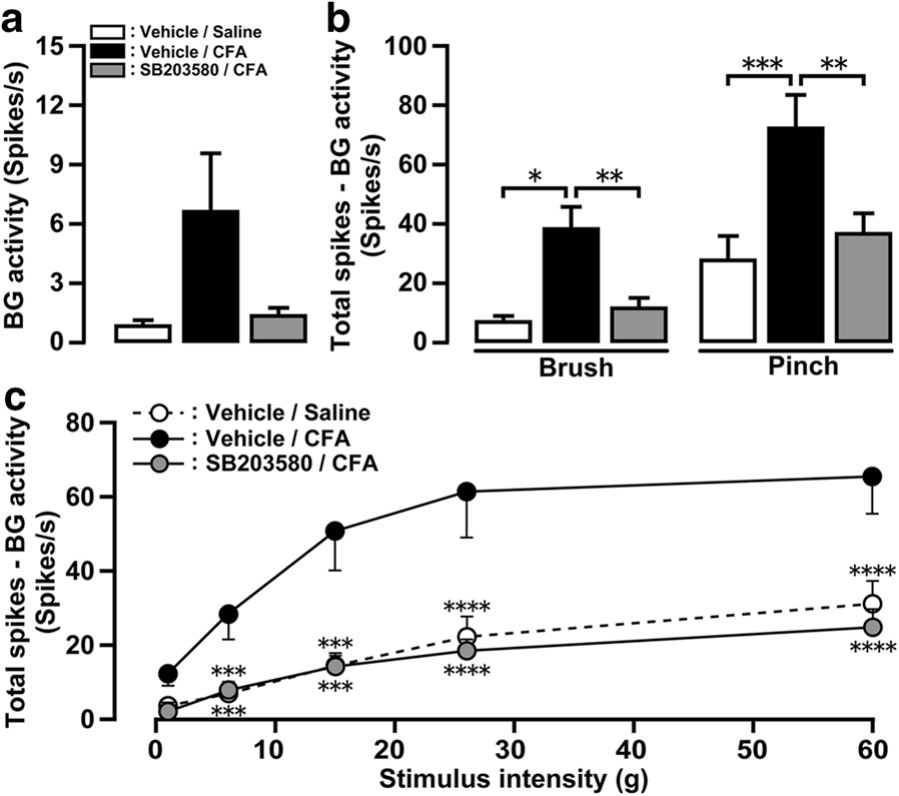
Mechanical-evoked responses of Vc neurons after CFA injection with i.c.m. SB203580 administration. **a** Background activities of WDR neurons. WDR neuronal responses to mechanical stimuli by brush, pinch stimuli (**b**) or von Frey filament (**c**) on day 4 after saline or CFA injection with i.c.m. vehicle or SB203580 administration (9 WDR neurons from 4 CFA-injected rats with i.c.m. vehicle administration, 9 WDR neurons from 4 CFA-injected rats with i.c.m. SB203580 administration, 9 WDR neurons from 3 saline-injected rats with i.c.m. vehicle administration). Data represent mean ± SEM. *p < 0.05, **p < 0.01, ***p < 0.001, ****p < 0.0001; by one-way ANOVA followed by Tukey’s multiple-comparison tests or by two-way ANOVA with repeated measures followed by Tukey’s multiple-comparison tests.

On day 4, the activity of WDR neurons were recorded from the Vc in CFA- or saline-injected rats, and the effects of i.c.m. administration of SB203580 on WDR neuronal activity in CFA-injected rats were assessed. The activity of twenty-seven WDR neurons was recorded from Vc in CFA-injected rats with i.c.m. vehicle or SB203580 administration, and in saline-injected rats with i.c.m. vehicle administration. On day 4, we found no significant difference in the background activities after saline injection with i.c.m. vehicle administration, after CFA injection with i.c.m. vehicle administration or after CFA injection with i.c.m. SB203580 administration (Fig. 6a). Further, brush- and pinch-evoked responses in CFA-injected rats with i.c.m. vehicle administration were significantly larger than that of saline-injected rats with i.c.m. vehicle administration or CFA-injected rats with i.c.m. SB203580 administration (Fig. 6b). In CFA-injected rats, i.c.m. SB203580 administration significantly suppressed noxious as well as non-noxious mechanical-evoked responses of WDR neurons compared with the i.c.m. vehicle-administered group (Fig. 6c). All recorded WDR neurons were located in the superficial laminae of the Vc (data not shown).

## Discussion

Various inflammatory pain models in the orofacial region were developed by CFA injection into the temporomandibular joint [18], the whisker pad skin [19], the parotid gland [20], the tongue [21] or capsaicin injection into the lateral facial skin [22]. These models were assessed with mechanical or heat sensitivity on the inflammatory locus. On the other hand, it is well known that the patients who suffered from dental pain induced by pulp inflammation sometimes complain about the referred pain in other orofacial structures that have no pathological changes, which can account for misdiagnosis or inappropriate treatment [23, 24]. Therefore, it was necessary to clarify the mechanisms underlying ectopic orofacial pain associated with remote local inflammation by developing an appropriate animal model. In this study, mechanical allodynia in the lateral facial skin was induced and there were no pathological changes in the lateral facial skin on day 4 following trapezius muscle inflammation, indicating that mechanical allodynia in the orofacial region can be induced by local inflammation in an area remote from this region. Thus, the established orofacial ectopic pain model is likely to be especially useful in the clarification of the pathogenic mechanisms of orofacial ectopic pain [17].

Primary afferent neurons are hyperexcited due to peripheral inflammation including the orofacial region, which also facilitate the release of various neurotransmitters in parallel with neuromodulators. These include adenosine triphosphate (ATP), substance P, calcitonin gene-related peptide, or brain-derived neurotrophic factor from the central terminals of primary neuron, which in turn cause changes in the excitability of postsynaptic nociceptive neurons [25, 26]. In microglia, P2X receptor expression is restricted to the P2X_4_ and P2X_7_ receptor subtypes [27–29]. The extracellular application of ATP induces an intracellular Ca^2+^ elevation in microglia, the intracellular Ca^2+^ elevation which is induced by binding to intrinsic P2X_7_ receptors phosphorylates p38 [30, 31]. p38 is phosphorylated in activated microglia labeled with CD11b or Iba-1, and activated microglia demonstrates morphological changes e.g. hypertrophy or process retraction/extension [32–34]. We observed obvious microglial activation in Vc which had a large soma with thick processes associated with trapezius muscle inflammation, and p38 was phosphorylated in the activated microglia. Further, successive i.c.m. administration of SB203580 suppressed p38 phosphorylation in microglia and microglial activation in Vc. Our results suggest that microglial activation accompanied with morphological changes in Vc and C1-C2 is induced via p38 phosphorylation following trapezius muscle inflammation. Though p38 phosphorylation in microglia was potentially facilitated by ATP signaling via P2X_7_ receptor, further studies to determine upstream signals of p38 phosphorylation in microglia attributable to peripheral inflammation are needed.

In this study, it was confirmed that the successive i.c.m. administration of SB203580 completely abolished p38 phosphorylation in Vc following trapezius muscle inflammation. The successive i.c.m. administration of SB203580 also completely depressed the mechanical allodynia in the lateral facial skin, and microglial activation in Vc following trapezius muscle inflammation. The mechanical-evoked responses of WDR neurons in Vc were significantly enhanced after trapezius muscle inflammation, and the evoked responses showed an increase in their firings following an increase in mechanical stimulus intensity. The successive i.c.m. administration of SB203580 depressed the enhancement of the mechanical-evoked responses. Further, the enhanced background activity, and brush- and pinch-evoked responses in WDR neurons after trapezius muscle inflammation were also suppressed by the successive i.c.m. administration of SB203580. Moreover, IL-1β was significantly over-expressed in microglia in Vc ipsilateral to the inflamed trapezius muscle, and the successive i.c.m. administration of SB203580 completely depressed the IL-1β protein over-expression. It is well established that p38 phosphorylation in microglia accelerates the synthesis of proinflammatory cytokines such as TNF-α, IL-1β, and IL-6, and these cytokines are released in the medulla or spinal cord [16]. Together, these findings suggest that microglial activation followed by trapezius muscle inflammation is prerequisite for activation of IL-1β post-translational maturation, and plays a critical role for the induction of IL-1β-converting enzyme activity and IL-1β release.

**Fig. 7:**
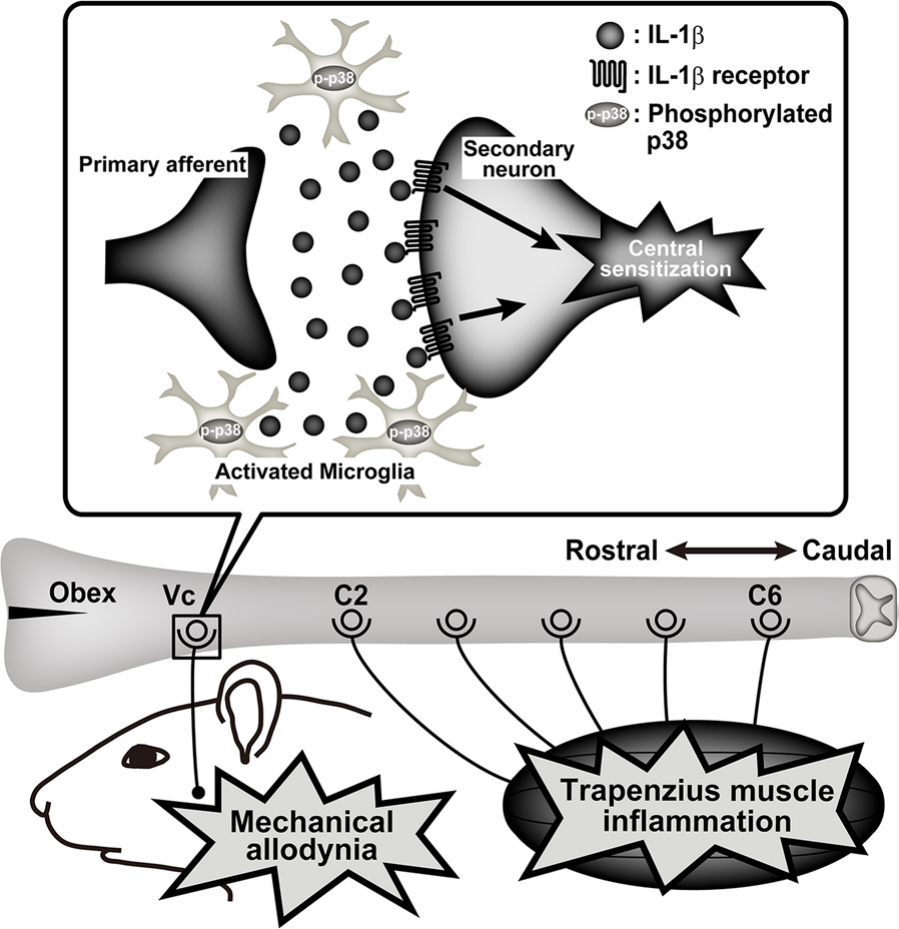
Schematic presentation of contribution of p38 phosphorylation in medullary microglia to ectopic orofacial inflammatory pain. Microglia in medulla and upper cervical spinal cord was activated following trapezius muscle inflammation. IL-1β release from the activated microglia in trigeminal spinal subnucleus caudalis (Vc) was accelerated through p38 phosphorylation and the excitability of wide dynamic range (WDR) neurons in Vc was enhanced via IL-1β signaling. The enhancement of excitability of WDR neurons in Vc after trapezius muscle inflammation may result in ectopic mechanical allodynia in orofacial region.

Intrathecal administration of IL-1β induces both thermal and mechanical hypersensitivity due to changes in spinal dorsal horn (SDH) neuronal responses to noxious stimuli [35, 36]. The IL-1 receptor type I (IL-1RI), which is known as a functional receptor of IL-1β, functions as the ligand binding subunit in the IL-1 receptor complex [37]. IL-1RI exists in SDH neurons localized in the superficial layers of the SDH, which suggests that IL-1β signaling in SDH neurons influence neuronal excitability [38]. Indeed, intrathecal IL-1β administration increases the wind-up activity of SDH neurons [39]. IL-1β signaling also increases in intracellular Ca^2+^ concentration in SDH neurons, resulting in facilitation of the excitatory neurotransmission in the SDH [40–42]. Moreover, intrathecal IL-1β administration inhibits both the frequency and the amplitude of spontaneous postsynaptic currents in SDH neurons, reducing both gamma-aminobutyric acid- and glycine-mediated currents, indicating that the increase of IL-1β is also capable of facilitating excitatory neurotransmission by suppressing inhibitory neurotransmission [43]. Taken together, the results of this study suggest that the increased release of IL-1β from activated microglia via p38 phosphorylation enhances neuronal excitability in Vc WDR neurons, which are involved in mechanical allodynia in the lateral facial skin associated with trapezius muscle inflammation (Fig. 7).

## Conclusion

WDR neuronal hyperexcitability in Vc is associated with an increase in IL-1β release from activated microglia via p38 phosphorylation, which plays a key role in developing the mechanical allodynia in the lateral facial skin following trapezius muscle inflammation. Thus, p38 phosphorylation in microglia may be a promising therapeutic target for treating ectopic pain associated with trapezius muscle pain.

## Methods

### Animals

All experiments were performed on male Sprague–Dawley rats (Japan SLC, Shizuoka, Japan) weighing 210–260 g (n = 230). All rats were kept under the climate-controlled room on a 12 h light/dark cycle (light on at 7:00, light off at 19:00) with food and water ad libitum, and individually housed in clear polycarbonate cages (length × width × height = 48 × 26.5 × 21 cm) containing wood shavings as bedding in a home cage. The number of rats used in this study was based on the minimum required for statistical analysis. In all experiments, the sequencing of surgery and testing was pseudo-randomized to control for any possible confounding effects of order of testing. All studies were conducted in strict accordance with the National Institutes of Health Guide for the Care and Use of Laboratory Animals and the guidelines of the International Association for the Study of Pain [44] and approved by the local animals ethics committee in Nihon University.

### Induction of inflammation in trapezius muscle

Initially, rats were deeply anesthetized with an intraperitoneal (i.p.) injection of sodium pentobarbital (50 mg/kg; Schering Plough, Whitehouse Station, NJ) and were placed on a warm mat (37 °C). The incision of the neck skin was made using the scalpel. 50 % Complete Freund’s adjuvant (CFA; 90 μl, diluted in saline, Sigma-Aldrich, St. Louis, MO) or saline was injected into the trapezius muscle. The incised skin was sutured with 5–0 silk. After the surgery, benzyl penicillin potassium (20,000 units, Penicillin G potassium, Meiji Seika, Tokyo, Japan) was intramuscularly injected to prevent infection.

### Changes in mechanical sensitivity of the facial skin

Rats were allowed to acclimate for 1 h prior to observation in behavioral testing room, and placed in individual observation chambers. Rats were trained in daily sessions for approximately 5 days to keep quietly protruding their snout for 10 min from a hole in its front wall [45]. The rats could escape freely from mechanical stimulation to the facial skin under this condition without physical restraints. To assess changes in the mechanical sensitivity of the lateral facial skin ipsilateral to CFA or saline injection, von Frey filaments (Touch Test Sensory Evaluator, North Coast Medical, Morgan Hill, CA) were applied to the lateral facial skin before and after CFA or saline injection into trapezius muscle for 15 days. On day 4 after CFA injection into the trapezius muscle with i.c.m. continuous administration of SB203580, the mechanical sensitivity of facial skin was defined as described above. The filament was applied at 1 s intervals for each stimulation, and the head-withdrawal threshold (HWT) was defined as the lowest force required to elicit a head-withdrawal reflex more than 3 of 5 stimulations. All behavioral testings were conducted under blind conditions.

### Iba1 immunoreactivity in Vc

The rats were anesthetized with sodium pentobarbital (50 mg/kg, i.p.) and transcardially perfused with saline followed by a fixative containing 4 % paraformaldehyde (PFA) in 0.1 M phosphate buffer (PB; pH = 7.4) on days 4 and 15 after CFA or saline injection into the trapezius muscle. Then, the medulla was dissected out and fixed in 4 % PFA for 1 day at 4 °C, and after that kept in 0.01 M phosphate buffer saline (PBS) containing 20 % sucrose for overnight for cryoprotection. Thirty micrometer thick tissue sections were cut from the medulla using a freezing microtome (Leica, Tokyo, Japan), and every sixth sections were collected in 0.01 M PBS. After rinsing the free-floating tissue sections in 0.01 M PBS, the sections were incubated in rabbit polyclonal Iba1 antibody (1:2,000, Wako, Osaka, Japan) to define activated microglial cells, for 72 h at 4 °C following incubating in 10 % normal goat serum (NGS) in PBS for 1.5 h at room temperature (RT). Then, the sections were incubated in biotinylated goat anti-rabbit IgG (1:600, Vector Laboratories, Burlingame, CA, USA) for 2 h at RT. After rinsing with 0.01 M PBS, the sections were incubated in peroxidase-conjugated avidin–biotin complex (1:100, Vector Laboratories, Burlingame, CA) for 1 h at RT. After washing in 0.05 M Tris buffer (TB, pH 7.4), the sections were incubated in 0.035 % 3,3′-daiminobenzidine tetrahydrochloride hydrate (DAB, Sigma-Aldrich, St. Louis, MO), 0.2 % nickel ammonium sulfate, and 0.05 % peroxide in 0.05 M TB for about 5 min. After washing in PBS, the sections were serially mounted on MAS-coated Superfrost Plus microscope slides (Matsunami, Osaka, Japan), dehydrated in a series of ethanol (from 50 to 100 %), and coverslipped.

Iba1 expression was analyzed in a square grid (26.7 × 26.7 μm^2^) of Vc (1,440, 2,160 and 2,800 μm caudal to the obex) that receives afferents from the trigeminal nerve innervating the lateral facial skin. The area occupied by the Iba1 immuno-products was measured using a computer-assisted imaging analysis system (ImageJ 1.37v; NIH, Bethesda, MD).

Additionally, rats were deeply anesthetized with sodium pentobarbital (50 mg/kg, i.p.) and transcardially perfused with saline followed by a fixative containing 4 % PFA in 0.1 M PB on day 4 after CFA injection into the trapezius muscle with i.c.m. continuous administration of SB203580. Then, the area occupied by the Iba1 immuno-products in Vc was measured as described above after the medulla and upper cervical spinal cord were dissected out and fixed for 1 day at 4 °C and kept in 0.01 M PBS containing 20 % sucrose for overnight for cryoprotection.

We also performed immunohistochemical staining without primary antibody for Iba1 for three sections in each group of rats, and no immuno-products could be observed (data not shown).

### Double-labeling immunohistochemistry

Rats were anesthetized with sodium pentobarbital (50 mg/kg, i.p.) and transcardially perfused with saline followed by a fixative containing 4 % PFA in 0.1 M PB (pH 7.4) on day 4 after CFA or saline injection into the trapezius muscle. The medulla and upper cervical cord were kept in 0.01 M PBS containing 20 % sucrose for 12 h for cryoprotection following dissection after perfusion and immersed in the same fixative for 4 h at 4 °C. The medulla were embedded in TissueTek (Sakura Finetek, Tokyo, Japan) and cut using a cryostat at a thickness of 10 μm. The medulla and upper cervical cord sections were thaw-mounted on MAS-coated Superfrost Plus microscope slides. Then, they were dried at RT in a dark room overnight. After rinsing with 0.01 M PBS, the sections were incubated with mouse monoclonal phospho-p38 MAPK (Thr180/Tyr182) antibody (1:100, Cell Signaling) and rabbit polyclonal Iba1 antibody (1:1,000, Wako), rabbit polyclonal glial fibrillary acidic protein (GFAP) antibody (1:1,000, Dako) or rabbit polyclonal anti-neuronal nuclei (NeuN) antibody (1:100, Merck Millipore, Billerica, MA) in 0.01 M PBS containing 3 % NGS and 0.3 %Triton X-100 overnight at 4 °C to identify the localization of phospho-p38 MAPK in Vc. The sections were incubated with rabbit polyclonal anti-IL-1β antibody (1:100, Abcam, Cambridge, UK) and mouse monoclonal anti-Iba1 antibody (1:100, Abcam), mouse monoclonal anti-GFAP antibody (1:400, Merck Millipore) or mouse monoclonal anti-NeuN antibody (1:100, Merck Millipore) in 0.01 M PBS containing 3 % NGS and 0.3 % Triton X-100 overnight at 4 °C to identify the localization of IL-1β in Vc. After rinsing with 0.01 M PBS, the sections were incubated in Alexa Fluor 488 anti-mouse IgG (1:100; Invitrogen) and Alexa Fluor 568 anti-rabbit IgG (1:100; Invitrogen) in 0.01 M PBS for 2 h at RT. Following rinsing with 0.01 M PBS, the sections were coverslipped in mounting medium (Thermo Fisher Scientific, Waltham, MA) and double immunoreactive (IR) cells were analyzed using a fluorescence microscope (Keyence, Osaka, Japan).

### Western blotting analysis

The rats were anesthetized with sodium pentobarbital (50 mg/kg, i.p.) and perfused with saline on day 4 following CFA or saline injection into the trapezius muscle. The medulla and upper cervical cord were excised and immediately homogenized in 100 μl of ice-cold lysis buffer (137 mM NaCl, 20 mM Tris–HCl, pH 8.0, 1 % NP40, 10 % glycerol, 1 mM phenylmethylsulfonyl fluoride, 10 μg/ml aprotinin, 1 g/ml leupeptin, and 0.5 mM sodium vanadate) using a tube pestle (Thermo Fisher Scientific). Samples were centrifuged at 15,000 rpm for 10 min at 4 °C and the supernatant was collected. Following the protein concentration of the sample was determined with a protein assay kit (Bio-Rad, Hercules, CA, USA), the sample was heat-denatured in Laemmli sample buffer solution (Bio-Rad). The sample (30 μg) was subjected to electrophoresis for protein separation on 10 % SDS-PAGE and electroblotted onto polyvinylidene difluoride membranes (Trans-Blot turbo Transfer pack, Bio-Rad) by using Trans-Blot Turbo (Bio-Rad). The membrane was incubated overnight at 4 °C with phospho-p38 (pp38) MAPK (Thr180/Tyr182) antibody (1:1,000, Cell Signaling) diluted in Tris-Buffered Saline and Tween 20 (TBST) containing 5 % bovine serum albumin (BSA) following 3 % BSA (Bovogen, Essendon, Australia) incubation. After incubation with horseradish peroxidase (HRP)-conjugated donkey anti-rabbit antibodies (Cell Signaling), each protein binding was visualized by using Western Lightning ELC Pro (PerikinElmer, Waltham, MA, USA). Band intensity was quantified using a ChemiDoc MP system (Bio-Rad). After removing protein binding on the membrane using a stripping reagent (Thermo Scientific), the band intensity was normalized to p38 immunoreactivity on blots reproved with anti-p38 antibodies (1:1,000, Cell Signaling).

Furthermore, the rats were anesthetized with sodium pentobarbital (50 mg/kg, i.p.) and perfused with saline on day 4 after CFA injection into the trapezius muscle with i.c.m. continuous administration of SB203580. After the medulla and upper cervical spinal cord were excised and homogenized, the supernatant was collected. After that, the protein concentration of the sample was determined with a protein assay kit (Bio-Rad). The protein amounts of phospho-p38 (pp38) and IL-1β in the prepared sample were determined by the method described above (see “Western blotting analysis”) using pp38 MAPK (Thr180/Tyr182) antibodies (1:1,000, Cell Signaling) or anti-IL-1β antibodies (1:1,000, Abcam) as primary antibodies, and HRP-conjugated donkey anti-rabbit antibodies (Cell Signaling) as secondary antibodies. In turn, the normalization was performed using β-actin or p38 immunoreactivity on blots reproved with anti-β-actin antibody (1:200, Santa Cruz, Santa Cruz, CA, USA) or anti-p38 antibody (1:1,000, Cell Signaling). The p38 and β-actin immunoreactivities were used as a loading control.

### Inhibition of p38 phosphorylation in Vc

CFA or saline was injected into trapezius muscle under deep anesthesia with sodium pentobarbital (50 mg/kg, i.p.) and simultaneously placed in a stereotaxic frame. After each rat’s skull was exposed by midline skin incision, a small hole (diameter, 1 mm) was drilled above the location of the caudal part of the skull with a dental drill to insert a polyethylene tube (SP45; size: 0.5 × 0.8 mm; Natsume, Tokyo, Japan) into the cisterna magna [46]. A selective inhibitor of p38 MAPK, SB203580 (8 μmol/day, 10 % dimethyl sulfoxide dissolved in saline, Cell Signaling), was intra-cisterna magnally (i.c.m.) administrated for 4 successive days (day 0 through day 3) using a micro-osmotic infusion pump (Model 2001; Alzet Durect, Cupertino, CA, USA) connected the polyethylene tube.

### Vc neuronal recording

On day 4 after CFA or saline injection into the trapezius muscle in rats with i.c.m. continuous administration of SB203580 or vehicle, a single-neuron recording was performed in Vc neurons using the procedures described previously [47]. The trachea and right femoral vein were cannulated to allow artificial respiration and intravenous administration of drugs under deep anesthesia using sodium pentobarbital (50 mg/kg, i.p.). The anesthesia was maintained with isoflurane (2–3 %) mixed with oxygen during the experiment. The rat was placed in a stereotaxic frame, dura and pia mater were removed following laminectomy to expose brain surface. The rat was artificially ventilated with immobilization using pancuronium bromide (0.6 mg/kg, i.v.; Schering-Plough, Darmastadt, Germany). The electrocardiogram was monitored and the hearting rate was maintained at 250–300/min, End-tidal CO_2_ was maintained at 3.5–4.5 % and the rectal temperature at 37 °C by a feedback-controlled heating blanket (Nihon Koden, Tokyo, Japan). A pool was made with skin flaps around the laminectomy which was performed to expose Vc, and the brainstem kept wet with isotonic saline. A tungsten microelectrode (impedance = 13 MΩ, 1000 Hz, FHC, St Bowdin, ME) was inserted into Vc to record single neuronal activity. Based on their responses to innocuous or noxious mechanical stimulation to the lateral facial skin, the recorded neurons were classified either as low-threshold mechanoreceptive neurons, WDR neurons or nociceptive-specific (NS) neurons, as previously described [48]. In this study, only WDR neurons were analyzed. The activity of this single neuron was augmented using a differential amplifier (Nihon Koden) and stored in PC. Spike frequencies of stored single neuronal activity were analyzed using the Spike II software (CED 1401, Cambridge, UK). Background activity was first recorded for 30 s before application of mechanical stimulation to the facial skin following identification of WDR neurons. For mechanical stimulation of the receptive field, graded stimuli with von Frey filaments (1, 6, 15, 26 and 60 g) and brushing with a nylon-hair brush were applied for 5 s at 10 s intervals. High-intensity stimulation with pinch was produced by a small arterial clip. After neuronal recording, the recording sites in Vc were histologically identified as previously described [49].

### Statistical analysis

Data were expressed as mean ± SEM. Statistical analyses were performed by Student’s *t* test, one-way ANOVA followed by Tukey’s multiple-comparison tests, Kruskal–Wallis tests, two-way ANOVA followed by Bonferroni’s or Tukey’s multiple-comparison tests where appropriate. A value of *p* <0.05 was considered significant.

## Abbreviations

TMD: temporomandibular disorder
CFA: complete freund’s adjuvant
WDR: wide dynamic range
Vc: trigeminal spinal subnucleus caudalis
MAPK: mitogen-activated protein kinase
SB203580: 4-(4′-fluorophenyl)-2-(4′-methylsulfinylphenyl)-5-(4′-pyridyl)-imidazole
CNS: central nervous system
Iba1: ionized calcium-binding adaptor molecule-1
IL-1β: interleukin-1β
I.p.: intraperitoneal
HWT: head-withdrawal threshold
PFA: paraformaldehyde
PB: phosphate buffer
PBS: phosphate buffer saline
NGS: normal goat serum
RT: room temperature
TB: tris buffer
GFAP: glial fibrillary acidic protein
IR: immunoreactive
TBST: tris-buffered saline
BSA: bovine serum albumin
HRP: horseradish peroxidase
I.c.m.: intra-cisterna magnally
pp38: phospho-p38
ATP: adenosine triphosphate
NMDA: N-methyl-d-aspartate

## References

[1] Fernandez-de-Las-Penas C, Ge HY, Arendt-Nielsen L, Cuadrado ML, Pareja JA (2007). Referred pain from trapezius muscle trigger points shares similar characteristics with chronic tension type headache. Eur J Pain.

[2] Fernandez-de-Las-Penas C, Galan-Del-Rio F, Alonso-Blanco C, Jimenez-Garcia R, Arendt-Nielsen L, Svensson P (2010). Referred pain from muscle trigger points in the masticatory and neck-shoulder musculature in women with temporomandibular disoders. J Pain Off J Am Pain Soc..

[3] Marini I, Bartolucci ML, Bortolotti F, Gatto MR, Bonetti GA (2012). Palmitoylethanolamide versus a nonsteroidal anti-inflammatory drug in the treatment of temporomandibular joint inflammatory pain. J Orofac Pain..

[4] Turp JC, Kowalski CJ, Stohler CS (1997). Pain descriptors characteristic of persistent facial pain. J Orofac Pain..

[5] Svensson P, Cairns BE, Wang K, Hu JW, Graven-Nielsen T, Arendt-Nielsen L (2003). Glutamate-evoked pain and mechanical allodynia in the human masseter muscle. Pain.

[6] Svensson P, Castrillon E, Cairns BE (2008). Nerve growth factor-evoked masseter muscle sensitization and perturbation of jaw motor function in healthy women. J Orofac Pain..

[7] Koistinaho M, Koistinaho J (2002). Role of p38 and p44/42 mitogen-activated protein kinases in microglia. Glia..

[8] Turjanski AG, Vaque JP, Gutkind JS (2007). MAP kinases and the control of nuclear events. Oncogene.

[9] Zeng KW, Yu Q, Song FJ, Liao LX, Zhao MB, Dong X (2015). Deoxysappanone B, a homoisoflavone from the Chinese medicinal plant Caesalpinia sappan L., protects neurons from microglia-mediated inflammatory injuries via inhibition of IkappaB kinase (IKK)-NF-kappaB and p38/ERK MAPK pathways. Eur J Pharmacol.

[10] Yang C, Yu L, Kong L, Ma R, Zhang J, Zhu Q (2014). Pyrroloquinoline quinone (PQQ) inhibits lipopolysaccharide induced inflammation in part via downregulated NF-kappaB and p38/JNK activation in microglial and attenuates microglia activation in lipopolysaccharide treatment mice. PLoS One.

[11] Haraguchi K, Kawamoto A, Isami K, Maeda S, Kusano A, Asakura K (2012). TRPM2 contributes to inflammatory and neuropathic pain through the aggravation of pronociceptive inflammatory responses in mice. J Neurosci Off J Soc Neurosci.

[12] Schafer S, Berger JV, Deumens R, Goursaud S, Hanisch UK, Hermans E (2014). Influence of intrathecal delivery of bone marrow-derived mesenchymal stem cells on spinal inflammation and pain hypersensitivity in a rat model of peripheral nerve injury. J Neuroinflamm.

[13] Schilling T, Repp H, Richter H, Koschinski A, Heinemann U, Dreyer F (2002). Lysophospholipids induce membrane hyperpolarization in microglia by activation of IKCa1 Ca(2+)-dependent K(+) channels. Neuroscience.

[14] El-Remessy AB, Tang Y, Zhu G, Matragoon S, Khalifa Y, Liu EK (2008). Neuroprotective effects of cannabidiol in endotoxin-induced uveitis: critical role of p38 MAPK activation. Mol Vis..

[15] Svensson CI, Hua XY, Protter AA, Powell HC, Yaksh TL (2003). Spinal p38 MAP kinase is necessary for NMDA-induced spinal PGE(2) release and thermal hyperalgesia. NeuroReport.

[16] Ji RR, Suter MR (2007). p38 MAPK, microglial signaling, and neuropathic pain. Mol Pain..

[17] Kiyomoto M, Shinoda M, Okada-Ogawa A, Noma N, Shibuta K, Tsuboi Y (2013). Fractalkine signaling in microglia contributes to ectopic orofacial pain following trapezius muscle inflammation. J Neurosci.

[18] Shinoda M, Ozaki N, Asai H, Nagamine K, Sugiura Y (2005). Changes in P2X3 receptor expression in the trigeminal ganglion following monoarthritis of the temporomandibular joint in rats. Pain.

[19] Miyamoto M, Tsuboi Y, Honda K, Kobayashi M, Takamiya K, Huganir RL (2012). Involvement of AMPA receptor GluR2 and GluR3 trafficking in trigeminal spinal subnucleus caudalis and C1/C2 neurons in acute-facial inflammatory pain. PLoS One.

[20] Okada-Ogawa A, Shinoda M, Honda K, Iwata K (2012). New models of experimental parotitis and parotid gland distension in rats. Methods Mol Biol.

[21] Liu MG, Matsuura S, Shinoda M, Honda K, Suzuki I, Shibuta K (2012). Metabotropic glutamate receptor 5 contributes to inflammatory tongue pain via extracellular signal-regulated kinase signaling in the trigeminal spinal subnucleus caudalis and upper cervical spinal cord. J Neuroinflamm..

[22] Honda K, Kitagawa J, Sessle BJ, Kondo M, Tsuboi Y, Yonehara Y (2008). Mechanisms involved in an increment of multimodal excitability of medullary and upper cervical dorsal horn neurons following cutaneous capsaicin treatment. Mol Pain..

[23] Bender IB (2000). Pulpal pain diagnosis–a review. J Endod.

[24] Farella M, Michelotti A, Gargano A, Cimino R, Ramaglia L (2002). Myofascial pain syndrome misdiagnosed as odontogenic pain: a case report. Cranio J Craniomandib Prac..

[25] Woolf CJ, Salter MW (2000). Neuronal plasticity: increasing the gain in pain. Science.

[26] Chizh BA, Illes P (2001). P2X receptors and nociception. Pharmacol Rev.

[27] Tsuda M, Shigemoto-Mogami Y, Koizumi S, Mizokoshi A, Kohsaka S, Salter MW (2003). P2X4 receptors induced in spinal microglia gate tactile allodynia after nerve injury. Nature.

[28] Ferrari D, Villalba M, Chiozzi P, Falzoni S, Ricciardi-Castagnoli P, Di Virgilio F (1996). Mouse microglial cells express a plasma membrane pore gated by extracellular ATP. J Immunol..

[29] Kobayashi K, Yamanaka H, Noguchi K (2013). Expression of ATP receptors in the rat dorsal root ganglion and spinal cord. Anat Sci Int.

[30] Volonte C, Apolloni S, Skaper SD, Burnstock G (2012). P2X7 receptors: channels, pores and more. CNS Neurol Disord Drug Targets.

[31] Nakanishi M, Mori T, Nishikawa K, Sawada M, Kuno M, Asada A (2007). The effects of general anesthetics on P2X7 and P2Y receptors in a rat microglial cell line. Anesth Analg.

[32] Jin SX, Zhuang ZY, Woolf CJ, Ji RR (2003). p38 mitogen-activated protein kinase is activated after a spinal nerve ligation in spinal cord microglia and dorsal root ganglion neurons and contributes to the generation of neuropathic pain. J Neurosci Off J Soc Neurosci..

[33] Chen XY, Li K, Light AR, Fu KY (2013). Simvastatin attenuates formalin-induced nociceptive behaviors by inhibiting microglial RhoA and p38 MAPK activation. J Pain Off J Am Pain Soc..

[34] Ji RR, Berta T, Nedergaard M (2013). Glia and pain: is chronic pain a gliopathy?. Pain.

[35] Reeve AJ, Patel S, Fox A, Walker K, Urban L (2000). Intrathecally administered endotoxin or cytokines produce allodynia, hyperalgesia and changes in spinal cord neuronal responses to nociceptive stimuli in the rat. Eur J Pain.

[36] Sung CS, Wen ZH, Chang WK, Ho ST, Tsai SK, Chang YC (2004). Intrathecal interleukin-1beta administration induces thermal hyperalgesia by activating inducible nitric oxide synthase expression in the rat spinal cord. Brain Res.

[37] Boraschi D, Tagliabue A (2013). The interleukin-1 receptor family. Semin Immunol.

[38] Zhang RX, Li A, Liu B, Wang L, Ren K, Zhang H (2008). IL-1ra alleviates inflammatory hyperalgesia through preventing phosphorylation of NMDA receptor NR-1 subunit in rats. Pain.

[39] Constandil L, Hernandez A, Pelissier T, Arriagada O, Espinoza K, Burgos H (2009). Effect of interleukin-1beta on spinal cord nociceptive transmission of normal and monoarthritic rats after disruption of glial function. Arthritis Res Ther.

[40] Viviani B, Bartesaghi S, Gardoni F, Vezzani A, Behrens MM, Bartfai T (2003). Interleukin-1beta enhances NMDA receptor-mediated intracellular calcium increase through activation of the Src family of kinases. J Neurosci Off J Soc Neurosci..

[41] Salter MW, Pitcher GM (2012). Dysregulated Src upregulation of NMDA receptor activity: a common link in chronic pain and schizophrenia. FEBS J.

[42] Ren K, Torres R (2009). Role of interleukin-1beta during pain and inflammation. Brain Res Rev.

[43] Kawasaki Y, Zhang L, Cheng JK, Ji RR (2008). Cytokine mechanisms of central sensitization: distinct and overlapping role of interleukin-1beta, interleukin-6, and tumor necrosis factor-alpha in regulating synaptic and neuronal activity in the superficial spinal cord. J Neurosci Off J Soc Neurosci..

[44] Zimmermann M (1983). Ethical guidelines for investigations of experimental pain in conscious animals. Pain.

[45] Kitagawa J, Takeda M, Suzuki I, Kadoi J, Tsuboi Y, Honda K (2006). Mechanisms involved in modulation of trigeminal primary afferent activity in rats with peripheral mononeuropathy. Eur J Neurosci.

[46] Terayama R, Omura S, Fujisawa N, Yamaai T, Ichikawa H, Sugimoto T (2008). Activation of microglia and p38 mitogen-activated protein kinase in the dorsal column nucleus contributes to tactile allodynia following peripheral nerve injury. Neuroscience.

[47] Tsuboi Y, Iwata K, Dostrovsky JO, Chiang CY, Sessle BJ, Hu JW (2011). Modulation of astroglial glutamine synthetase activity affects nociceptive behaviour and central sensitization of medullary dorsal horn nociceptive neurons in a rat model of chronic pulpitis. Eur J Neurosci.

[48] Iwata K, Imai T, Tsuboi Y, Tashiro A, Ogawa A, Morimoto T (2001). Alteration of medullary dorsal horn neuronal activity following inferior alveolar nerve transection in rats. J Neurophysiol.

[49] Suzuki I, Tsuboi Y, Shinoda M, Shibuta K, Honda K, Katagiri A (2013). Involvement of ERK phosphorylation of trigeminal spinal subnucleus caudalis neurons in thermal hypersensitivity in rats with infraorbital nerve injury. PLoS One.

